# Case Report: Multisystem response to dupilumab in a child with atopic dermatitis, asthma, eosinophilic esophagitis, and other gastrointestinal manifestations

**DOI:** 10.3389/falgy.2026.1929466

**Published:** 2026-09-18

**Authors:** Hua-Jie Yan, Wei Dong, Yu-Nan Yang, Chun-Yun Zeng

**Affiliations:** Department of Pediatrics, Nanxiang Hospital of Jiading District, Shanghai, China

**Keywords:** asthma, atopic dermatitis, child, dupilumab, eosinophilic esophagitis, gastrointestinal eosinophilia, type 2 inflammation

## Abstract

**Background:**

Atopic dermatitis (AD), asthma, and eosinophilic esophagitis (EoE) may coexist because they share type 2 inflammatory pathways. Evidence for dupilumab in children with EoE and other gastrointestinal manifestations remains limited.

**Case presentation:**

A 7-year-old girl had poorly controlled AD, recurrent asthma, EoE (>100 eosinophils/high-power field), and lower-density eosinophilic infiltration in gastric, ileal, and colonic biopsies. These extraesophageal findings did not uniformly satisfy proposed thresholds for definite non-EoE eosinophilic gastrointestinal disease. Severe AD despite topical therapy was the primary indication for dupilumab; the coexisting type 2 inflammatory disorders supported selection of a pathway-directed systemic treatment. Dupilumab was administered as a 600-mg loading dose followed by 300 mg subcutaneously every 4 weeks. Over 48 weeks, pruritus resolved, skin lesions markedly improved, wheezing did not recur, inhaled salmeterol/fluticasone was reduced, appetite improved, and vomiting ceased. Follow-up endoscopy showed a smooth esophagus and no obvious duodenal abnormality, although gastric inflammation and small ulcers persisted and repeat histology was unavailable. Total IgE decreased from >2,000 to 211.2 IU/mL; peripheral eosinophils decreased from 0.64 to 0.30 × 10⁹/L. No treatment-related adverse events occurred.

**Conclusion:**

This case suggests that IL-4/IL-13 blockade may provide coordinated clinical benefit across multiple type 2 inflammatory organs. The gastrointestinal findings should be interpreted cautiously because symptom improvement was not confirmed by repeat biopsy.

## 1 Introduction

Atopic disorders frequently cluster rather than occurring in a fixed linear sequence. This pattern, described as atopic multimorbidity, reflects shared epithelial-barrier abnormalities and type 2 immune pathways across the skin, respiratory tract, and gastrointestinal tract (1, 2). Eosinophilic gastrointestinal diseases (EGIDs) comprise eosinophilic esophagitis (EoE) and non-EoE disorders affecting the stomach, small intestine, or colon. Patients with EGIDs commonly have coexisting allergic disease, including asthma and AD (3–5).

Dupilumab is a fully human IgG4 monoclonal antibody directed against the interleukin-4 receptor alpha subunit and inhibits signaling by both IL-4 and IL-13. Its established effects in AD and type 2 asthma, together with randomized evidence in EoE and emerging reports in non-EoE EGIDs, provide a mechanistic rationale for its use when several type 2 inflammatory disorders coexist (6–12). We report the clinical course of a child with AD, asthma, EoE, and extraesophageal eosinophilic gastrointestinal involvement who received dupilumab for 48 weeks.

## 2 Case description

### 2.1 Dermatologic presentation

A 7-year-old girl (weight, 21 kg; height, 108 cm) presented to the pediatric clinic with a more than 4-year history of generalized pruritic dermatitis. Her father had dermatographism; her mother had no reported allergic disease. Examination showed scattered erythematous papules, scaling, excoriations, hemorrhagic crusts, and lichenification over the neck, trunk, and extremities (Figures 1A–D). Chart-recorded baseline assessments were SCORAD 104, investigator's global assessment (IGA) 3, combined itch/sleep numerical rating score (NRS) 12, and Dermatology Life Quality Index (DLQI) 11. Because the recorded SCORAD and combined NRS exceed the conventional maxima for the standard SCORAD and single-item 0–10 NRS instruments, these values are presented exactly as documented in the clinical record and should be interpreted cautiously. The adult DLQI was used in the source clinical record rather than the age-appropriate Children's Dermatology Life Quality Index (CDLQI), which is validated for children aged 4–16 years. Therefore, the recorded DLQI values are reported descriptively and should also be interpreted cautiously. Total serum IgE was >2,000 IU/mL, with sensitization predominantly to house dust mites and additional animal-dander, pollen, and food sensitization (Table 1). Intermittent desonide ointment, topical tacrolimus, and oral cetirizine had provided inadequate control.

**Figure 1:**
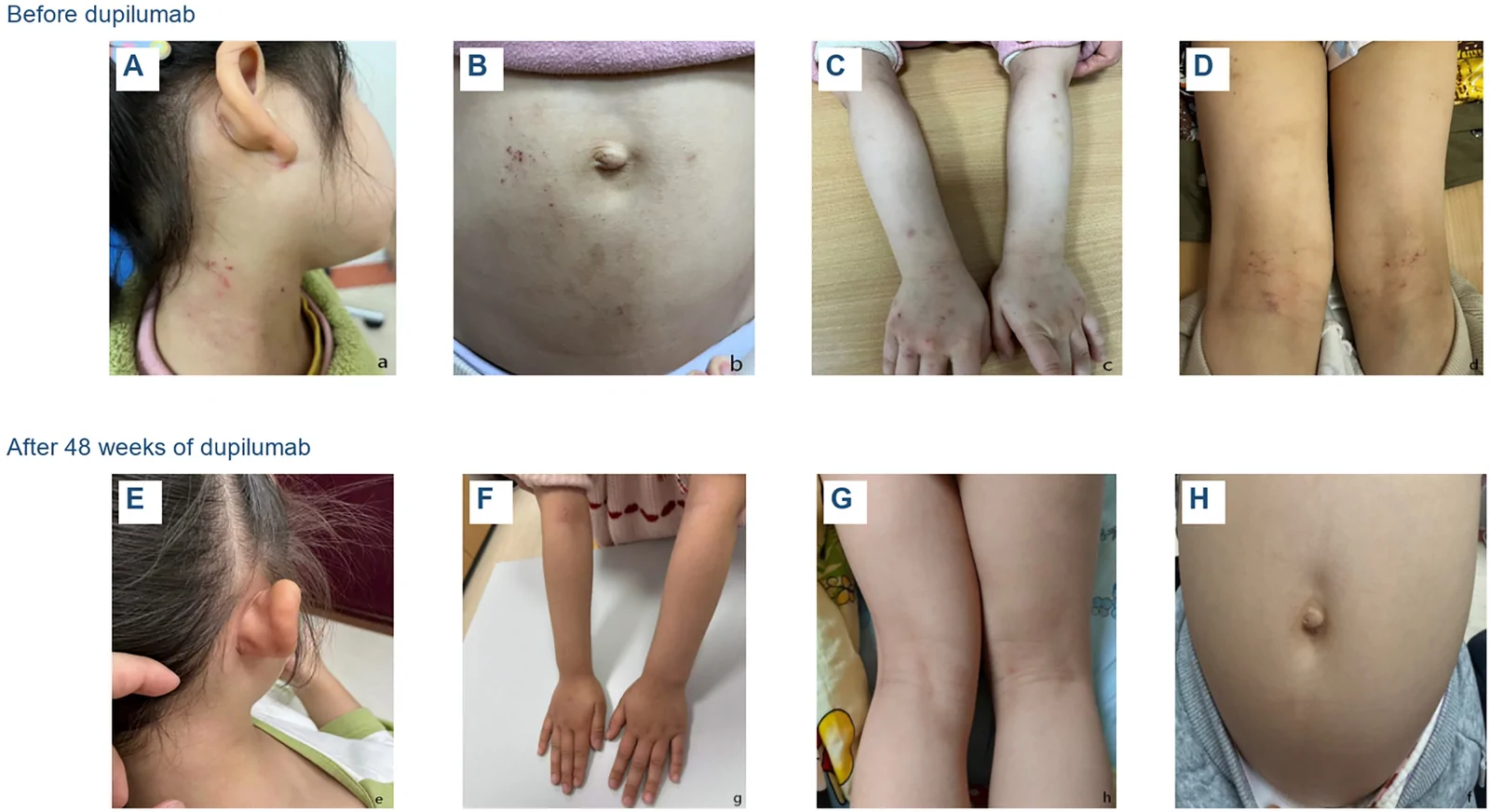
Clinical photographs before and after dupilumab treatment. Before treatment, papules, erythema, scaling, excoriation, and lichenification were present behind the ear, on the abdomen, and on the extremities **(A–D)**. After 48 weeks, all photographed sites showed marked improvement, with residual erythema, textural change, or post-inflammatory change visible at some sites **(E–H)**. Images were obtained during routine clinical care rather than under fully standardized photographic conditions. Written permission for publication of the clinical images was obtained from the patient's legal guardian.

**Table 1:** Serum allergen-specific IgE before and after dupilumab treatment.

Analyte	22 May 2024	14 Apr 2026
Dermatophagoides pteronyssinus	20.7	0.73
Dermatophagoides farinae	>100	53.7
Blomia tropicalis	>100	19.2
Cat dander	24.2	0.94
Dog dander	3.3	0.04
Short ragweed	1.5	0
Humulus scandens	0.57	0
Chenopodium/Amaranthus retroflexus	3.6	0
Juniper/birch	0.6	0
Plane tree/ash	1.1	0
June grass/ryegrass/timothy grass	0.5	0
Poplar/willow/beech/oak/walnut	2.3	0
Boxelder/mulberry/black locust/elm/cypress/paper mulberry	2.1	0
Egg yolk	3.9	0
Egg white	0.63	0
Milk	0.48	0.50
Peanut/soybean	4.5	0
Sesame	0.05	0
Wheat/buckwheat	0.27	0
Cashew/pistachio/hazelnut/almond/walnut	0.02	0
Peach/apple/mango/lychee/strawberry	0.07	0
Total IgE (IU/mL)	>2,000	211.2

Allergen-specific IgE values are reported in kU/L; total IgE is reported in IU/mL. The pre-treatment total IgE exceeded the assay's upper limit.

### 2.2 Respiratory and gastrointestinal history

The first wheezing episode occurred on December 5, 2020, followed by approximately five to six episodes per year. Acute asthma exacerbations required hospitalization in August and October 2022; treatment included intravenous methylprednisolone, cefuroxime, and nebulized budesonide, ipratropium, and salbutamol. On August 30, 2024, fractional exhaled nitric oxide was 13 ppb and bronchodilator testing was positive, with a 23.2% improvement in forced expiratory volume in 1 s (FEV1), representing a positive bronchodilator response suggestive of reversible airflow limitation in the context of the clinical history of asthma. Maintenance salmeterol/fluticasone (50 μg per inhalation, twice daily) was prescribed.

On August 27, 2024, she was evaluated for intermittent vomiting that had persisted for approximately 1 year. Upper endoscopy on August 28 showed mild squamous epithelial hyperplasia of the esophagus and focal antral erosion. Biopsies contained >100 eosinophils/high-power field (HPF) in the esophagus and 5–10 eosinophils/HPF in the stomach. Ileocolonoscopy showed focal lymphoid hyperplasia in the terminal ileum and sigmoid colon; biopsy eosinophil counts were 2–8/HPF in the terminal ileum, 5–10/HPF in the sigmoid colon, and 20–30/HPF in the transverse colon. Peripheral eosinophils were 0.75 × 10⁹/L (12.0%). The esophageal findings supported EoE. The number and anatomical levels of esophageal biopsies and a formal EoE Endoscopic Reference Score were not recorded in the available retrospective record. The lower-density extraesophageal eosinophilia did not uniformly meet proposed thresholds for definite non-EoE EGID and is therefore described as gastrointestinal eosinophilic infiltration rather than a confirmed multi-segment EGID. Gastric erosions and the duodenal-bulb ulcer were considered nonspecific and required clinicopathological correlation. Intravenous omeprazole and methylprednisolone were given during the acute episode in August 2024. The overall clinical course and the major diagnostic and therapeutic events are summarized in Figure 3.

## 3 Diagnostic assessment, therapeutic intervention, follow-up, and outcomes

### 3.1 Therapeutic rationale and regimen

The primary indication for systemic biologic therapy was persistent severe AD despite topical corticosteroid, calcineurin-inhibitor, and antihistamine treatment. The coexisting asthma and EoE supported selection of a treatment targeting the shared IL-4/IL-13 pathway. After the legal guardian provided informed consent, dupilumab was started on February 27, 2025, with a 600-mg loading dose followed by 300 mg subcutaneously every 4 weeks. Fourteen injections were administered during the reported period.

### 3.2 Atopic dermatitis response

Improvement became prominent from week 16. The chart-recorded SCORAD values at weeks 0, 8, 16, 32, and 48 were 104, 89, 36, 27, and 27, respectively; corresponding IGA values were 3, 3, 2, 2, and 1. Combined itch/sleep NRS values were 12, 12, 4, 2, and 1, and chart-recorded DLQI values were 11, 12, 6, 3, and 3 (Figures 2A,B). Because the adult DLQI was used instead of the age-appropriate CDLQI, these values were not treated as an age-validated pediatric outcome measure. By week 48, pruritus had resolved and skin lesions were markedly improved (Figures 1E–H). Topical anti-inflammatory treatment was stopped; cetirizine drops were used intermittently.

**Figure 2:**
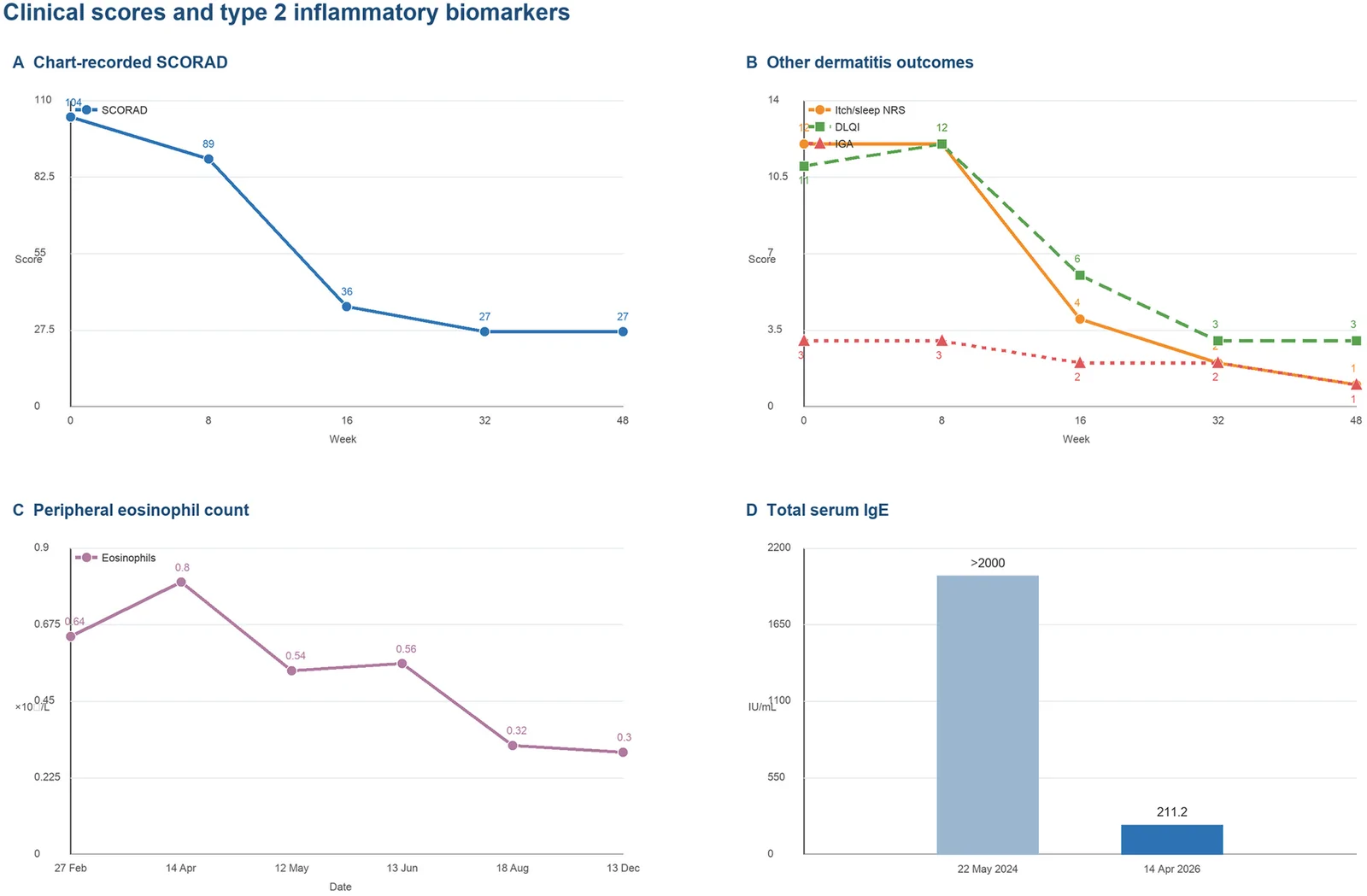
Clinical scores and biomarkers during follow-up. **(A)** Chart-recorded SCORAD. **(B)** Combined itch/sleep NRS, DLQI, and IGA, distinguished by different colors, line patterns, and point markers. **(C)** Peripheral eosinophil count from the day dupilumab was started through December 2025. **(D)** Total serum IgE before and after treatment. The baseline total IgE was above the assay upper limit (>2,000 IU/mL) and is plotted at 2,000 for visualization. Values in panels A and B are reproduced exactly from the clinical record; interpretation caveats are described in the text. The DLQI values were obtained using the adult instrument rather than the age-appropriate CDLQI and are presented descriptively. CDLQI, Children's Dermatology Life Quality Index; DLQI, Dermatology Life Quality Index; IGA, investigator's global assessment; NRS, numerical rating score; SCORAD, SCORing Atopic Dermatitis.

**Figure 3:**
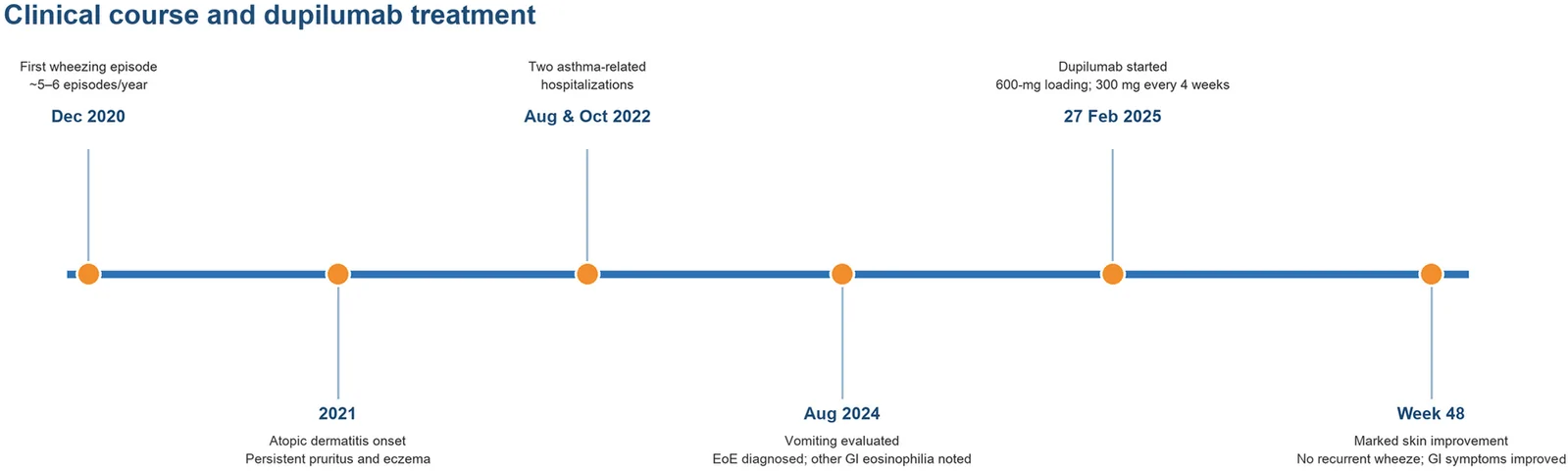
Timeline of the clinical course, diagnostic evaluation, dupilumab treatment, and outcomes. AD, atopic dermatitis; EoE, eosinophilic esophagitis; GI, gastrointestinal.

### 3.3 Asthma response

No recurrent wheezing was reported during follow-up, although occasional cough persisted. On December 27, 2025, spirometry was normal: FEV1 was 108.5% of predicted and FEV1/FVCmax was 103.6% of predicted. Salmeterol/fluticasone was progressively reduced to one inhalation every 2 days. The absence of a complete baseline spirometry dataset and the continued, although reduced, inhaled controller therapy preclude attributing respiratory improvement to dupilumab alone.

### 3.4 Gastrointestinal response

Intravenous omeprazole and methylprednisolone had been administered during the August 2024 gastrointestinal episode, approximately 6 months before dupilumab initiation; oral omeprazole was subsequently continued. The exact treatment durations and the degree of gastrointestinal symptom improvement before dupilumab were not fully documented. Follow-up upper endoscopy on August 19, 2025, approximately 24 weeks after dupilumab initiation, showed a smooth esophageal mucosa and no obvious duodenal papillary or mucosal abnormality. However, the fundus and antrum remained congested and edematous, with erosions and multiple small antral ulcers. Appetite improved and abdominal pain, distension, and vomiting were absent. Because corticosteroid and proton-pump inhibitor treatment preceded or accompanied dupilumab and repeat mucosal biopsies were unavailable, histological remission and the independent gastrointestinal effect of dupilumab cannot be established.

### 3.5 Biomarker response

Total IgE decreased from >2,000 IU/mL on May 22, 2024, to 211.2 IU/mL on April 14, 2026, and multiple allergen-specific IgE values also decreased (Table 1). Peripheral eosinophils were 0.64 × 10⁹/L (7.8%) immediately before dupilumab and 0.30 × 10⁹/L (2.9%) on December 13, 2025 (Figures 2C,D). Eosinophil counts fluctuated during early treatment, reaching 0.80 × 10⁹/L on April 14, 2025, before subsequently declining. No treatment-related adverse events or treatment interruptions occurred during the 48-week follow-up.

## 4 Discussion

This case documents improvement across three organ systems during 48 weeks of dupilumab treatment in a child with marked atopic multimorbidity. The most robust evidence is the dermatologic response, supported by serial clinician- and patient-reported scores, clinical photographs, withdrawal of topical anti-inflammatory therapy, and a substantial fall in total and allergen-specific IgE. Respiratory control also improved, with no recurrent wheeze, normal follow-up spirometry, and reduction—but not discontinuation—of inhaled salmeterol/fluticasone.

IL-4 and IL-13 contribute to epithelial-barrier dysfunction, IgE class switching, eosinophilic inflammation, and airway hyperresponsiveness, providing a common mechanistic basis for benefit across AD, asthma, and EoE (2, 6–9). In the phase 3 trial reported by Dellon et al., weekly dupilumab improved histological and symptomatic outcomes in adolescents and adults with EoE (8). A case report described rapid improvement in coexisting EoE and allergic asthma (9). For non-EoE EGIDs, retrospective series and pediatric reports have described symptomatic and histological improvement in eosinophilic gastritis, duodenitis, gastroenteritis, and colitis (10–12). These observations support considering pathway-directed therapy as a unified, or “umbrella,” strategy when several type 2 inflammatory diseases coexist.

The gastrointestinal course in this patient was mixed. The esophageal eosinophil density supported EoE, whereas the gastric, ileal, and colonic counts did not uniformly meet proposed thresholds for definite non-EoE EGID. Accordingly, the extraesophageal findings are interpreted as gastrointestinal eosinophilic infiltration and associated manifestations rather than confirmed multi-segment EGID. Symptoms resolved and the esophageal and duodenal appearance improved, while gastric inflammation and ulcers persisted. Other causes of the nonspecific gastric and duodenal lesions cannot be excluded. Because corticosteroid and proton-pump inhibitor therapy preceded or accompanied dupilumab and repeat biopsy was not performed, the case supports a possible multisystem clinical benefit but does not demonstrate dupilumab-induced gastrointestinal histological remission.

## 5 Strengths and limitations

Strengths include 48 weeks of follow-up, serial dermatitis scores, objective pulmonary-function reassessment, repeated endoscopy, longitudinal eosinophil measurements, allergen-specific IgE testing, and clinical photographs. Limitations include the single-patient retrospective design; concomitant therapies; incomplete baseline spirometric values; use of the adult DLQI instead of the age-appropriate CDLQI in a 7-year-old patient; unavailable numbers and anatomical levels of esophageal biopsies; absence of validated EoE symptom and endoscopic scoring, including EREFS; uncertainty in classifying the lower-density extraesophageal eosinophilia; absence of a fully documented differential assessment and follow-up gastrointestinal histology; non-standardized clinical photography; and uncertainty regarding two chart-recorded dermatology measures that exceeded conventional scale maxima. Changes in IgE and eosinophils may indicate biological modulation of type 2 inflammation but are not established biomarkers of clinical response, particularly in EGIDs; organ-specific clinical outcomes remain the primary evidence of therapeutic effect.

## Patient perspective

The patient's legal guardian reported that relief of itching, wheezing, vomiting, and poor appetite substantially reduced the child's symptom burden and simplified daily treatment. The family was satisfied with the overall 48-week outcome.

## Data Availability

The original contributions presented in the study are included in the article/Supplementary Material, further inquiries can be directed to the corresponding author.
